# The double burden of malnutrition: coexistence of stunted children and overweight and obese mothers in Sri Lanka

**DOI:** 10.1017/S1368980026102742

**Published:** 2026-06-01

**Authors:** Gayathri Abeywickrama, Sabu S. Padmadas, Andrew Hinde

**Affiliations:** 1 Faculty of Health and Wellbeing, University of Winchester, UK; 2 Social Statistics and Demography, University of Southampton, UK; 3 Southampton Statistical Sciences Research Institute, University of Southampton, UK

**Keywords:** Double burden of malnutrition, Child stunting, Overweight, Obesity

## Abstract

**Objective:**

Sri Lanka faces a double burden of malnutrition (DBM), characterised by the persistence of child undernutrition alongside rising rates of obesity and non-communicable diseases among women. This study aimed to quantify the prevalence of DBM and identify specific household, maternal and child-level risk factors associated with stunted child–overweight/obese mother pairs in Sri Lanka.

**Design:**

Data from the 2016 Sri Lanka Demographic and Health Survey were used to assess the risk of double-burden pairs through multinomial logistic regression, adjusting for individual, socio-economic and geographic factors. The primary outcome measures were child stunting, classified according to height-for-age *Z*-scores, and maternal BMI. Households with a stunted child and an overweight or obese mother were classified as having a DBM.

**Setting:**

A nationally representative sample covering all geographic regions of Sri Lanka.

**Participants:**

5975 mother–child pairs, comprising children aged 6–59 months and mothers aged 15–49 years with complete anthropometric data.

**Results:**

Overall, 8·3 % of households had a stunted child with an overweight mother, while 2·8 % had a stunted child and an obese mother. Child’s age, low birth weight, maternal age, household size, mode of delivery, wealth status and province of residence were significantly associated with the double burden among child–mother pairs (*P* < 0·001).

Ethnicity was also significant, with Muslim mothers more likely to reside in households experiencing DBM (*P* < 0·001).

**Conclusion:**

DBM poses a critical nutritional challenge in Sri Lanka, necessitating comprehensive, integrated interventions targeting both child stunting and maternal overweight to improve health outcomes and reduce long-term disease risk at the household and population levels.

Malnutrition, in its various forms, coexists at global, regional, country and household levels. Globally, nearly one in every three persons suffers from at least one form of malnutrition^(1)^. In 2022, approximately 2·5 billion adults were overweight worldwide, including 890 million who were obese^(2)^. In addition, about 390 million adults were underweight. In the same year, among children aged under 5 years, 37 million were found to be overweight or obese, while 149 million were affected by stunting^(3)^. The double burden of malnutrition (DBM) refers to the coexistence of undernutrition alongside overweight and obesity and can manifest in different forms. DBM may occur at the population level, where undernutrition and overweight coexist within the same setting; at the household level, where undernourished and overweight individuals live in the same household; or at the individual level, where different forms of malnutrition coexist within the same person across the life course.

The prevalence of DBM and its impact on diet-related non-communicable diseases (NCD) across the life course is a global public health concern. DBM contributes to diet-related NCD through the coexistence of early-life undernutrition and later exposure to energy-dense, nutrient-poor diets, which together increase metabolic vulnerability, insulin resistance and the risk of obesity, diabetes, CVD and other NCD in later life^(2–4)^.

Growing evidence highlights substantial nutrition transitions in low- and middle-income countries (LMIC), characterised by shifts from traditional diets to more energy-dense and processed foods, rapid changes in population age structure and an increasing burden of NCD alongside persistent undernutrition^(5,6)^. These transitions involve rapid changes in dietary habits, lifestyles and broader socio-economic conditions^(4,7)^, contributing to a global rise in obesity and NCD. Notably, health disparities are widening, disproportionately affecting poor and marginalised groups.

LMIC face a dual malnutrition crisis characterised by a coexistence of persistent undernutrition in infants and children and rising overnutrition in adults. These intergenerational challenges are perpetuated by poor maternal nutrition, which increases the likelihood of low birth weight (LBW) infants and contributes to the vicious cycles of malnutrition^(4,6)^. LBW children face higher risks of stunting and, paradoxically, obesity later in life, reinforcing the prevalence of stunted child–overweight mother (SCOWT) pairs^(8,9)^. Conversely, maternal obesity elevates risks of gestational diabetes, macrosomia and offspring obesity^(10)^. This phenomenon, termed *paradoxical malnutrition*, occurs when households exhibit both undernutrition (wasting and stunting) and overnutrition (overweight and obesity)^(7)^.

Dual-burden households, particularly those with overweight mothers and stunted children, are becoming a critical public health concern in LMIC^(11)^. The Developmental Origins of Health and Disease (DOHaD) hypothesis suggests that nutritional and environmental exposures during critical windows of early development, from conception through early childhood, can lead to lasting physiological adaptations that influence growth, metabolic function and susceptibility to chronic disease risks throughout the life course^(12,13)^. These implications extend beyond individuals, affecting societal health and economic stability. Stunted children may experience impaired cognitive development, reduced productivity and chronic health issues, while obesity increases susceptibility to NCD – hypertension, diabetes, CVD and cancer-straining healthcare systems^(14–16)^.

Recognising the urgency, the WHO advocates for integrated approaches to tackle the DBM, aligning with Sustainable Development Goal 2. The proposed ‘double-duty actions’ aim to simultaneously address undernutrition and overnutrition, offering a holistic approach to global malnutrition challenges^(17,18)^. Sri Lanka is confronted with a significant public health challenge posed by the DBM^(19)^. Despite improvements in health indicators such as maternal health and life expectancy, child undernutrition rates have remained stagnant, with 17 % of children stunted, 15 % wasted and 21 % underweight over the last two decades^(20)^. Concurrently, the prevalence of maternal overweight and obesity has nearly doubled, with 32 % of women classified as overweight and 13 % as obese^(21)^.

Despite implementing interventions such as the National Nutrition Policy (2010), Multi-Sector Action Plan for Nutrition (2013–2016) and various food supplementation and poverty alleviation programmes, Sri Lanka has seen minimal improvement in child nutrition indicators^(20–22)^. Rapid nutritional, epidemiological and socio-economic transitions have significantly altered household nutritional practices, with a shift towards including energy-dense, nutrient-poor diets, further exacerbating malnutrition^(5,23,24)^. The ongoing health, demographic and social transitions in Sri Lanka have increased life expectancy but also accelerated the rise of NCD, which account for 83 % of all deaths, with nearly one in five occurring prematurely; adult obesity has emerged as a significant contributing risk factor within this mortality profile^(25,26)^.

Limited national-level data exist on the DBM, particularly on child–mother pairs, highlighting an urgent need for studies to investigate the factors contributing to the coexistence of child undernutrition and maternal overnutrition and the socio-economic determinants of this dual burden^(19,27)^. Addressing these gaps is critical for developing targeted interventions to mitigate the DBM and its associated health and social consequences in Sri Lanka. This research investigates the influence of individual, maternal, household and residential factors on the DBM, specifically the coexistence of child stunting and maternal overweight and obesity within the same household. It offers key insights to inform targeted household-level intervention strategies for addressing the DBM.

Despite Sri Lanka’s ongoing burden of malnutrition, the coexistence of undernourished children and overweight mothers, as well as the converse pattern, has not previously been systematically examined. The present study addresses this critical evidence gap by providing the first comprehensive national assessment of the DBM across Sri Lanka using nationally representative Demographic and Health Survey data.

## Methods

Data for this study are drawn from the nationally representative Sri Lanka Demographic and Health Survey (SLDHS) conducted in 2016–2017. The SLDHS followed a two-stage stratified cluster sampling design, through which 28 720 housing units were selected and 27 210 households were successfully interviewed. Within these households, 18 510 eligible ever-married women aged 10–49 years were identified, of whom 18 302 completed the individual interview (response rate: 98·9 %). The present analysis was restricted to women aged 15–49 years, owing to the small number of respondents aged 10–14 years.

The analytical sample was derived from the SLDHS children’s recode file. A total of 8104 children aged 0–59 months from 7072 mothers aged 15–49 years from across 6642 households were initially identified. Children aged 0–5 months (*n* 712) were excluded, resulting in 7392 singleton children aged 6–59 months from 6550 mothers. Further, a total of 575 cases were excluded due to missing height-for-age data (*n* 320), missing maternal BMI information (*n* 57) or maternal pregnancy beyond 3 months (*n* 198), as pregnancy may affect the accuracy of BMI measurements. This yielded 6817 eligible child–mother pairs. For mothers with more than one child aged 6–59 months, only the youngest child was included to reflect the closest nutritional relationship^(9)^, yielding a final analytical sample of 5975 child–mother pairs (online Supplementary Figure 1).

Data were collected using standardised DHS questionnaires adapted for Sri Lanka and pre-tested in Sinhala, Tamil and English. Trained interviewers administered the surveys following DHS protocols. Anthropometric measurements of mothers and children aged 6–59 months were obtained by trained health officers using calibrated equipment, with quality control procedures throughout. Detailed survey instruments and validation procedures are reported in the SLDHS 2016–2017 final report.

### Outcome variable

There is no consistent definition of the DBM at the household level. Stunting was considered an indicator of child undernutrition status, and BMI was a widely used indicator to measure maternal nutritional status^(8,9,28)^.

Child stunting was chosen over wasting and underweight as it reflects chronic malnutrition caused by prolonged nutritional deficiencies, leading to impaired linear growth. Child undernutrition remains a persistent challenge in Sri Lanka (Jayawardena, 2020; The World Bank, 2016). Stunting was defined as a height-for-age *Z*-score below –2 sd (WHO, 2006). Maternal BMI was classified per WHO guidelines: underweight (< 18·5 kg/m^2^), normal weight (18·5–24·9 kg/m^2^), overweight (25–29·9 kg/m^2^) and obesity (≥ 30 kg/m^2^) (WHO, 2019).

### Explanatory variables

The explanatory variables were grouped into four categories: child, maternal, household and geographic/residential. Variables considered for children included age (recorded in months and categorised 6–11, 12–47 and 48–59 months), birth interval (first birth, less than 24 months, 24–47 months and 48 months and more), sex (male and female) and LBW status (normal weight and LBW (babies weighing less than 2500 g). Children’s current breast-feeding status and morbidity status (incidence of diarrhoea or cough or fever) for the 2 weeks preceding the survey were also included in the analysis.

Additionally, children’s current breast-feeding status and morbidity status were considered. Maternal variables included maternal age (15–20, 21–29, 30–34 and 35–49 years), mode of child delivery (normal or caesarean), marital status, highest level of completed education (up to and including grade 5, grade 6 to O/L (Ordinary Level), passed GCE Advanced Level (A/L) and Degree and above) and maternal employment status. Maternal height was defined by three categories: short (≤ 145 cm), average (145·1–155 cm) and tall (> 155 cm). Given the importance of maternal height as an indicator of intergenerational health and long-term nutritional status, maternal height was included as an independent variable alongside maternal BMI. Reliance on BMI alone may obscure associations related to maternal stature, as BMI does not fully capture the chronic nutritional and biological influences reflected in adult height. Multicollinearity between maternal height and BMI was assessed using variance inflation factors. The variance inflation factor values ranged from 1·8 to 2·1, indicating no meaningful collinearity and supporting the inclusion of both variables in the model.

Household variables included household size (recoded as < 5 members, 5–7 members and 8 and more) and wealth index. The study used the standard DHS wealth index quintiles. The wealth index is a composite measure of household asset ownership, housing materials and access to basic services, calculated using principal component analysis based on nationally representative household characteristics^(21,29)^.

Residential factors included sectors, divided into three sectors: urban, rural and estate. The urban sector is composed of areas administered by municipal and urban councils, whereas the estate sector is predominantly concentrated in the tea plantation areas, and the rural sector comprises the areas not captured by the urban and estate sectors within the nine administratively defined provinces^(30)^.

### Statistical analysis

In the initial analysis, the total sample of 5975 mother–child pairs was examined according to maternal BMI categories. Table 1 presents the bivariate associations between maternal BMI status and socio-economic characteristics (Table 1). A subsample of 3470 pairs, excluding non-stunted children with underweight, overweight or obese mothers, was considered for further bivariate analysis (Table 2). The Maternal and Child Double Burden (MCDB) was defined using five categories: (a) stunted child–underweight mother, (b) stunted child–normal-weight mother, (c) SCOWT, (d) stunted child–obese mother (SCOB) and (e) non-stunted child–normal-weight mother. SCOWT and SCOB pairs represented the DBM group.

**Table 1.** Association between child, maternal, household and residential factors and maternal BMI (*n* 5975) Table 1 long description.

	Thin (*n* 712)	Normal (*n* 2913)	Overweight (*n* 1709)	Obese (*n* 641)	All (5975)	*P*-value
Variables	%	%	%	%	%	
Child stunting						
Not stunted	11·1	48·4	29·2	11·2	4859	< 0·001
Stunted	15·4	50·1	25·8	8·7	1116	
Child age in months						
6–11 months	10·9	49·7	27·3	11·9	713	< 0·001
12–47 months	12·7	48·8	28·1	10·1	4140	
48–59 months	9·5	47·5	3·09	11·9	1122	
Birth interval						
First birth	16·4	49·8	25·8	7·7	2119	< 0·001
< 24 months	8·3	47·8	27·4	16·3	324	
24–47 months	10·6	48·7	28·7	11·8	1257	
48 months and more	8·8	47·8	31·2	12	2275	
Sex						
Male	11·4	49·9	28·3	10·3	3068	0·21
Female	12·4	47·4	28·9	11·1	2907	
Birth weight status						
Normal weight	10·5	48·5	29·6	11·2	4825	< 0·001
Low birth weight	18·4	50·2	23·5	7·8	987	
Missing					163	
Current breast-feeding						
Yes	13·1	50·0	27·5	9·2	3686	< 0·001
No	10·0	46·4	30·1	13·2	2233	
Missing					56	
Child morbidity						
Not had all	11·9	49·0	27·8	11·2	4408	0·06
Had one of the diseases	11·8	48·1	30·7	9·3	1559	
Missing					8	
Maternal age (years)						
15–20	25·7	52·0	17·3	4·8	225	< 0·001
21–29	15·1	49·3	26·2	9·1	2874	
30–34	7·9	49·2	30·7	12·0	1781	
35–49	6·9	45·6	33·5	13·8	1095	
Marital status						
Currently married/living together	11·9	49·0	28·7	11·0	5865	0·28
Widowed/divorced/separated	11·8	57·2	21·8	9·0	110	
Maternal education						
Up to grade 5	17·9	49·6	20·9	11·4	306	< 0·001
Grade 6 to Ordinary Level	12·6	49·1	27·8	10·3	4006	
Advanced Level	9·6	48·2	30·4	11·6	1345	
Degree and above	6·2	45·6	37·4	10·6	34	
Missing					284	
Maternal employment status						
Employed	11·9	49·9	27·7	10·2	1395	0·72
Not employed	11·8	48·3	28·8	10·8	4580	
Delivery mode						
Normal delivery	13·5	51·4	26·5	8·7	4158	< 0·001
Caesarean or other	8·3	43·4	32·7	15·5	1799	
Household size						
< 5 members	12·3	50·3	38·6	8·8	2436	< 0·001
5–7 members	11·2	48·3	28·8	11·8	3114	
8 members and more	15·3	43·5	27·1	14·1	425	
Wealth index						
Lowest	17·5	52·5	22·6	7·2	1504	< 0·001
Second	15·0	49·0	26·8	9·0	1216	
Middle	10·9	47·5	3·0	11·4	1138	
Fourth	8·0	48·1	31·9	11·7	1154	
Highest	4·9	44·6	34·2	16·2	963	
Residential sector						
Urban	8·5	41·9	33·1	16·3	949	< 0·001
Rural	11·7	49·7	28·5	10	4665	
Estate	23·5	54·0	17·7	4·7	361	
Ethnicity						
Sinhala	11·9	49·9	28·6	9·7	3946	< 0·001
Sri Lankan Tamil	12·3	49·6	26·6	11·0	1211	
Indian Tamil	21·2	55·4	18·4	4·8	184	
Muslims and others	8·3	37·0	36·7	17·8	634	
Province						
Western	8·7	45·5	31·7	14·0	1150	< 0·001
Central	12·3	52·2	26·7	8·6	751	
Southern	15·9	51·0	24·7	8·3	696	
Northern	10·1	48·3	29·5	11·9	713	
Eastern	9·9	47·7	29·9	12·3	664	
North-Western	12·3	46·3	30·5	10·8	665	
North-Central	12·4	48·7	26·6	12·1	402	
Uva	11·5	54·6	26·7	7·1	408	
Sabaragamuwa	17·3	48·1	27·0	7·6	526	

Values are % (*n*). *P*-values are from Pearson’s χ^2^ test. Missing observations were excluded from the χ^2^ tests.

**Table 2.** Percentage of child–mother pairs by demographic and socio-economic characteristics with χ^2^ tests of association (*n* 3470) Table 2 long description.

Covariates	Stunted child–underweight mother	Stunted child–normal-weight mother	Stunted child–overweight mother (SCOWT)	Stunted child–obese mother (SCOB)	Non-stunted child–normal-weight mother	*n*	*P*-value
Total cases	172	559	288	97	2354		
Overall %	4·9	16·1	8·3	2·8	67·8		
Child age in months							
6–11 months	2·6	15·4	10·1	1·4	70·2	414	< 0·001
12–47 months	5·8	17·1	8·6	3·0	65·3	2454	
48–59 months	2·8	12·4	5·8	2·6	76·2	602	
Birth interval							
First birth	6·5	13·9	6·4	2·0	71·0	1244	< 0·001
< 24 months	4·1	18·1	10·3	5·1	62·1	193	
24–47 months	4·3	16·6	9·7	3·1	66·2	740	
48 months and more	3·8	17·6	8·9	3·0	66·5	1293	
Sex							
Male	4·6	17·5	8·3	2·5	66·9	1815	0·12
Female	5·3	14·5	8·2	3·0	68·8	1655	
Birth weight status							
Normal weight	3·4	13·3	7·8	2·7	72·5	2725	< 0·001
Low birth weight	11·2	27·4	10·2	3·0	48·1	657	
Missing						88	
Child morbidity							
Not had all	5·1	46·6	8·5	2·8	66·7	2589	0·22
Had one of the diseases	4·4	14·3	7·5	2·6	71·0	624	
Missing						257	
Breast-feeding status							
Still breast-feeding	5·3	17·2	8·8	2·4	66·1	2211	0·06
Not breast-feeding	4·3	13·9	7·2	3·5	70·9	1222	
Missing						37	
Maternal age (years)							
15–20	10·4	11·8	5·5	2·1	69·9	143	< 0·001
21–29	6·4	16·3	7·6	2·2	67·2	1696	
30–34	2·8	15·9	8·3	3·2	69·6	1025	
35–49	3·1	16·6	10·5	3·8	65·8	606	
Maternal height							
Short <= 145 cm	7·5	33·2	13·7	6·5	38·9	277	< 0·001
Average 145·1–155 cm	5·8	18·2	9·8	2·8	63·1	1992	
Tall 155·1 and over	2·7	8·5	4·5	1·8	82·3	1201	
Maternal education							
Up to grade 5	10·4	27·2	7·3	2·6	52·3	191	< 0·001
Grade 6 to Ordinary Level	5·4	16·8	8·7	3·0	65·9	2376	
Advanced Level	2·7	11·5	7·1	2·1	76·4	738	
Degree and above	1·8	13·3	7·8	2·4	74·5	165	
Maternal marital status							
Currently married/living together	4·8	15·9	8·4	2·7	67·9	3398	
Widowed/separated/divorced	8·3	25·0	0·0	4·1	62·5	72	0·05
Maternal employment status							
Employed	5·8	17·5	6·2	2·7	68·3	811	< 0·001
Not employed	4·8	15·6	8·9	2·8	67·6	2659	
Delivery mode							
Normal	5·3	15·8	7·7	2·0	69·0	2495	< 0·001
Caesarean or other	3·8	16·9	9·7	4·9	64·5	956	
Missing						19	
Household factors							
Household size							
< 5 members	5·0	14·3	8·7	1·9	69·8	1454	< 0·001
5–7 members	4·7	17·3	7·7	3·1	67·1	1780	
8 members and more	5·9	17·8	10·7	5·5	60·5	236	
Wealth index							
Lowest	8·8	20·0	9·1	1·7	60·2	984	< 0·001
Second	5·2	17·0	8·4	3·4	65·6	720	
Middle	4·2	15·5	7·1	2·7	70·3	630	
Fourth	2·2	14·4	7·0	3·3	72·9	636	
Highest	1·2	9·8	9·4	3·4	76·2	500	
Ethnicity							
Sinhalese	4·4	14·6	7·4	2·3	71·1	2298	< 0·001
Sri Lankan Tamil	5·7	17·5	8·4	2·8	65·3	730	
Indian Tamil	14·8	30·7	8·1	1·4	45·1	135	
Muslims and others	2·6	17·2	14·0	6·8	59·2	307	
Residential factors							
Residential sector							
Urban	3·5	12·1	8·9	4·3	70·9	479	< 0·001
Rural	4·3	15·8	8·1	2·6	68·9	2737	
Estate	13·7	26·7	8·2	1·1	50·0	254	
Province							
Western	3·0	13·5	8·7	3·7	70·9	620	< 0·001
Central	7·5	23·3	10·0	2·0	56·9	488	
Southern	5·9	12·8	4·4	2·2	74·6	406	
Northern	3·9	7·4	7·6	3·4	67·4	406	
Eastern	3·1	16·4	9·9	3·9	66·4	382	
North-Western	6·2	12·4	8·6	1·8	70·8	370	
North-Central	3·4	11·9	9·4	3·4	71·9	234	
Uva	5·0	18·5	6·9	1·9	67·5	259	
Sabaragamuwa	6·5	17·3	8·5	1·9	65·5	305	

Values are % (*n*). *P*-values are from Pearson’s χ^2^ test. Missing observations were excluded from the χ^2^ tests.

Multinomial logistic regression was applied to assess the risk of MCDB relative to other maternal–child nutrition pairs. Results are reported as adjusted relative risk ratios (RRR) with 95 % CI. Table 3 presents the results of the fully adjusted multinomial logistic regression models (model 3) for child–mother pairs, adjusted for child, maternal, household and residential characteristics. Variables that were statistically significant in the bivariate analyses (*P* < 0·05) were included in the adjusted models to control for potential confounding. Adjusted RRR are reported only for associations that remained statistically significant (*P* < 0·05) in the final models. DBM categories involving stunted children were compared with non-stunted children with normal-weight mothers as the reference group, and the models assessed the independent associations of predictors with DBM outcome. Sampling weights were applied across all analyses. All statistical analyses were conducted using R statistical software (version 4.2.1).

**Table 3.** Adjusted relative risk ratios (aRRR) and 95 % CI for the final multinomial regression models of child–mother nutritional status pairs, adjusted for child, maternal, household and geographic factors Table 3 long description.

Covariate and category	Stunted child– underweight mother		Stunted child– normal-weight mother		Stunted child– overweight mother		Stunted child–obese mother	
	aRRR	95 % CI	aRRR	95 % CI	aRRR	95 % CI	aRRR	95 % CI
Intercept	0·10	0·29, 0·37	0·14	0·66, 0·32	0·07	0·02, 0·21	0·01	0·00, 0·04
Child factors								
Age in months								
6–11 months	1·00				1·00			
12–47 months	2·79	1·27, 4·63**			0·92	0·63, 1·35		
48–59 months	1·00	0·43, 2·31			0·49	0·30, 0·83**		
Birth weight status								
Normal	1·00		1·00		1·00		1·00	
Low birth weight	3·83	2·70, 5·43***	2·60	2·07, 3·27***	1·81	1·32, 2·47***	1·52	1·10, 2·20*
Maternal age (years)								
15–20					1·00			
21–29					1·59	0·74, 3·43		
30–34					1·92	0·87, 4·20		
35–49					2·32	1·05, 5·15*		
Maternal height								
Short	1·99	1·16, 3·14*	2·61	1·90, 3·59***	2·12	1·40, 3·22***	3·51	1·93, 6·40***
Normal	1·00		1·00		1·00		1·00	
Tall	0·45	0·30, 0·69***	0·41	0·32, 0·52***	0·36	0·26, 0·50***	0·47	0·28, 0·80**
Maternal education								
Up to grade 5	1·00		1·00		1·00			
Grade 6 to Ordinary Level	0·76	0·41, 1·39	0·79	0·53, 1·18	1·49	0·79, 2·81		
Advanced Level	0·67	0·31, 1·46	0·61	0·37, 0·98*	1·07	0·52, 2·19		
Degree and above	0·65	0·15, 2·52	0·77	0·34, 1·24	1·15	0·47, 2·81		
Maternal employment status								
Employed								
Not employed								
Delivery mode								
Normal							1·00	
Caesarean or other							2·43	1·56, 3·79***
Household factors								
Household size								
< 5 members			1·00				1·00	
5–7 members			1·21	1·02, 1·44*			1·67	1·02, 2·67*
8 members and more			1·31	0·87, 1·96			3·28	1·59, 6·78**
Wealth index								
Lowest	1·00		1·00		1·00		1·00	
Second	0·62	0·39, 0·98*	0·94	0·70, 1·27	0·78	0·53, 1·15	2·09	1·03, 4·11*
Middle	0·49	0·27, 0·78**	0·92	0·66, 1·28	0·66	0·43, 1·03	1·66	0·75, 3·70
Fourth	0·25	0·13, 0·48***	0·83	0·58, 1·17	0·61	0·39, 0·97*	1·80	0·81, 3·99
Highest	0·18	0·07, 0·49***	0·70	0·44, 1·09	1·03	0·62·1·71	2·05	0·82, 5·14
Ethnicity								
Sinhalese	1·00		1·00		1·00		1·00	
Sri Lankan Tamil	1·28	0·64, 2·57	1·00	0·66, 1·52	1·38	0·82, 2·34	1·32	0·56, 3·10
Indian Tamil	1·86	0·77, 4·48	1·52	0·80, 2·86	1·21	0·47, 3·15	1·58	0·22, 10·9
Muslims and others	0·94	0·42, 2·09	1·51	1·05, 2·11*	2·46	1·60, 3·77***	3·26	1·68, 6·30***
Ethnicity								
Sinhalese	1·00		1·00		1·00		1·00	
Sri Lankan Tamil	1·28	0·64, 2·57	1·00	0·66, 1·52	1·38	0·82, 2·34	1·32	0·56, 3·10
Indian Tamil	1·86	0·77, 4·48	1·52	0·80, 2·86	1·21	0·47, 3·15	1·58	0·22, 10·9
Muslims and others	0·94	0·42, 2·09	1·51	1·02, 2·11*	2·46	1·60, 3·77***	3·26	1·68, 6·30***
Residential factors								
Residential sector								
Urban	1·00		1·00		1·00		1·00	
Rural	0·65	0·39, 1·29	1·12	0·79, 1·57	0·94	0·63, 1·41	0·84	0·52, 1·71
Estate	0·72	0·31, 1·93	1·21	0·65, 2·24	0·64	0·28, 1·46	0·52	0·96, 2·99
Province								
Western	1·00		1·00		1·00		1·00	
Central	1·38	0·71, 2·74	1·46	1·01, 2·12*	1·32	0·83, 2·10	0·73	0·32, 1·66
Southern	1·68	0·82, 3·09	0·83	0·54, 1·20	0·49	0·27, 0·88*	0·60	0·24, 1·31
Northern	0·59	0·22, 1·45	1·21	0·66, 1·87	0·66	0·33, 1·31	1·47	0·44, 3·44
Eastern	0·57	0·22, 1·31	1·10	0·70, 1·68	0·89	0·52, 1·53	0·95	0·39, 1·97
North-Western	1·72	0·80, 3·12	0·79	0·51, 1·18	0·99	0·60, 1·63	0·50	0·18, 1·25
North-Central	0·93	0·35, 2·12	0·79	0·44, 1·19	1·03	0·58, 1·81	1·05	0·41, 2·38
Uva	0·74	0·32, 1·69	1·00	0·65, 1·55	0·74	0·41, 1·35	0·58	0·21, 1·66
Sabaragamuwa	1·14	0·57, 2·41	0·90	0·63, 1·47	0·82	0·47, 1·44	0·63	0·25, 1·75

^*^ *P* < 0·05; **P < 0·01; ***P < 0·001.

The variables retained in the final adjusted models for each child–mother pairs are included. Variables not statistically significant (*P* ≥ 0·05) after full adjustment were excluded from the final model and are therefore not presented. Non-stunted children with normal-weight mothers (Category 1) were designated as the reference group.

## Results

### Descriptive analysis

Table 1 presents the distribution of maternal BMI across child, maternal, household and residential characteristics among 5975 mother–child pairs. Among these mother–child pairs, the mean maternal BMI was 24·05 kg/m^2^. Overall, 11·9 % of mothers were underweight, 28·6 % were overweight and 10·7 % were obese, while 18·7 % of children were stunted (data not shown). Child characteristics, including stunting, age, birth interval, birth weight and breast-feeding status, were significantly associated with maternal BMI categories (*P* < 0·001). Although stunting was more prevalent among children of thin mothers, a notable proportion of stunted children were born to overweight (25·8 %) and obese (8·7 %) mothers, indicating the coexistence of maternal overnutrition and child undernutrition.

Among thin mothers, 18·4 % had LBW children, whereas among overweight (29·6 %) and obese (11·2 %) mothers, a higher proportion had normal-weight children. The prevalence of overweight (30·1 %) and obesity (13·2 %) was higher among non-breast-feeding mothers compared with breast-feeding mothers.

Maternal background characteristics, except marital status and employment status, were significantly associated with BMI. Overweight (33·5 %) and obesity (13·8 %) were most prevalent among older mothers aged 35–49 years. Among highly educated mothers, 37·4 % were overweight and 10·6 % were obese. Maternal BMI was also significantly linked to mode of delivery, with caesarean or other deliveries more frequent among overweight (32·7 %) and obese mothers (15·5 %).

Household size significantly influenced maternal BMI, with larger households showing higher obesity rates (14·1 %). BMI was also associated with household wealth: underweight (17·5 %) and normal-weight (52·5 %) mothers were more represented in the poorest group, while overweight (34·2 %) and obesity (16·2 %) were highest in the richest group.

Maternal BMI levels varied by residential sector, ethnicity and province. Overweight (33·1 %) and obesity (16·3 %) were highest among urban mothers, while underweight was most prevalent in the estate sector (23·5 %). Muslim mothers had the highest rates of overweight (36·7 %) and obesity (17·8 %), whereas underweight was most common among Indian Tamil mothers (21·2 %). Regionally, overweight (31·7 %) and obesity (14 %) were most common in the Western Province, while thinness was highest in the Sabaragamuwa Province (17·3 %).

**Figure 1.**
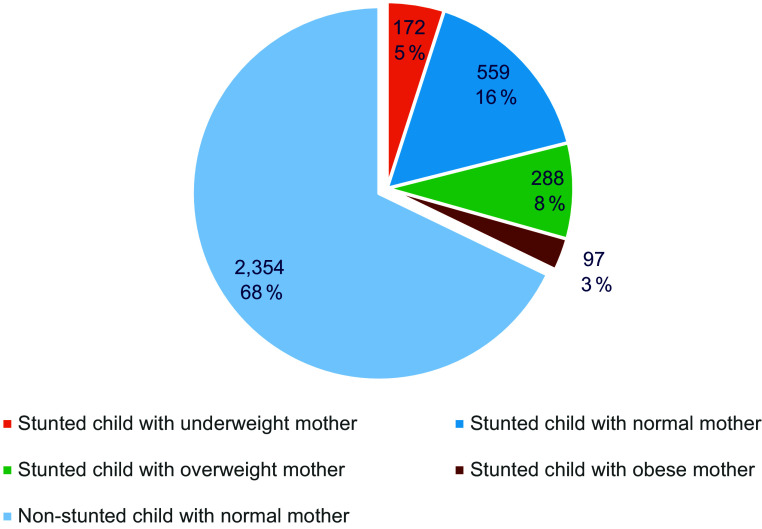
Figure 1 long description. Distribution (%) of total sample by child stunting status and maternal BMI (n = 3,470).

Figure 1 shows child–mother pairs based on child stunting and maternal BMI. Among the pairs, 68 % were non-stunted child–normal-weight mother and 16·1 % were stunted child–normal-weight mother. Double-burden pairs included 8·3 % SCOWT and 2·8 % SCOB. Additionally, 5 % were stunted child–underweight mother pairs.

Table 2 presents the bivariate relationship between these mother–child pairs and background characteristics. Among child factors, child’s age, birth interval and birth weight have a statistically significant association with the child–mother pairs. For example, the SCOWT pairs vary from 10·1 % among children aged 6–11 months to 5·8 % among children aged 48–59 months. Stunted child–normal-weight mother pairs were more prevalent among infants with LBW (27·4 %) than those with normal birth weight (13·3 %).

Differences were also observed across maternal characteristics. Paradoxical child–mother pairs (stunted children with overweight or obese mothers) were higher among mothers aged 35 years and above compared with younger mothers. Short maternal stature showed a greater representation in both SCOWT and SCOB pairs (13·7 % and 6·5 %, respectively). In addition, the distribution of mother–child nutritional pairs differed by maternal education level. DBM was more prevalent among mothers with lower educational attainment, with the highest proportion of SCOWT/SCOB pairs found in those educated up to grade 5 (7·3 % SCOWT and 2·6 % SCOB), whereas the lowest proportion was observed among mothers with degree-level education or higher (7·8 % SCOWT and 2·4 % SCOB). Maternal employment status was also statistically significantly associated with DBM. SCOWT pairs were more prevalent among mothers who were not employed (8·9 %**)** than those who were employed (6·2 %).

Mode of delivery was significantly associated with the nutritional status of child–mother pairs, with SCOWT and SCOB pairs more frequently observed among caesarean or other deliveries (9·7 % and 4·9 %, respectively).

In terms of household factors, DBM pairs were more apparent among households with eight and more members (10·7 % and 5·5 %). The richest households also had the lowest proportion of stunted children with underweight mother pairs (1·2 %). In terms of ethnicity, stunted child dyads with underweight and normal-weight mothers are significantly more common among Indian Tamils than among other groups (14·8 % and 30·7 %, respectively). In contrast, DBM pairs are relatively most common among Muslims and other minorities including Malays and Burghers (14·0 % are SCOWT and 6·8 % are SCOB pairs).

These DBM pairs are more common in urban areas (8·9 % and 4·3 %), whereas stunted child–underweight mothers were most common in the estate sector (13·7 %). SCOWT pairs lived in the Central Province (10·0 %). The highest proportion of SCOB pairs (3·9 %) was found in the Eastern Province, where the majority of the population is Muslim. Child’s sex, child morbidity, breast-feeding status and maternal marital status were not associated with variations in the mother–child nutritional status pairs.

### Multinomial regression analysis – association between child, maternal, household and residential factors and final models of child–mother pairs

Table 3 present the results of the multinomial regression analysis for all final models of child–mother pairs, adjusted for child, maternal, household and residential factors. Among child factors, LBW was strongly associated with stunting across all DBM child–mother nutritional status categories. Compared with children born at a normal birth weight, those with LBW had higher RRR of belonging to DBM-related stunting categories: SCOWT (RRR = 1·81; 95 % CI: 1·32, 2·47) and SCOB pairs (RRR = 1·52; 95 % CI: 1·10, 2·20).

Advanced maternal age (35 years and above) had a higher risk of being a SCOWT pair, with a 2·32 times greater likelihood compared to the reference group, after adjusting for other variables (RRR = 2·32; 95 % CI: 1·05, 5·15). However, the wide CI suggests limited precision.

Maternal height was also to be found a critical determinant of DBM, with the mothers with short stature being at the highest risk (RRR = 3·51, 95 % CI: 1·93, 6·40). The mode of delivery was also a significant predictor for stunted child–obese mother pairs. The relative risk for caesarean or other delivery was 2·43 times higher compared to women who had a normal delivery (RRR: 2·43, 95 % CI: 1·56, 3·79).

Among household factors, larger household size (8+ members) significantly increased the risk of being in a SCOB pair (RRR: 3·28; 95 % CI: 1·59, 6·78). Stunted child–underweight mother pairs were less common across all wealth quintiles, while the risk of SCOB pairs was higher in the second wealth quintile (RRR: 2·09; 95 % CI: 1·03, 4·11). Ethnicity showed a statistically significant association with double-burden mother–child pairs; Muslim mothers were 3·26 times more likely than Sinhalese mothers to be in a DBM pair (RRR: 3·26; 95 % CI: 1·68, 6·30). Geographically, the Southern Province showed a significantly lower risk of DBM pairs compared with the Western Province (RRR = 0·49; 95 % CI: 0·27, 0·88), although regional effects were attenuated after full model adjustment.

**Figure 2.**
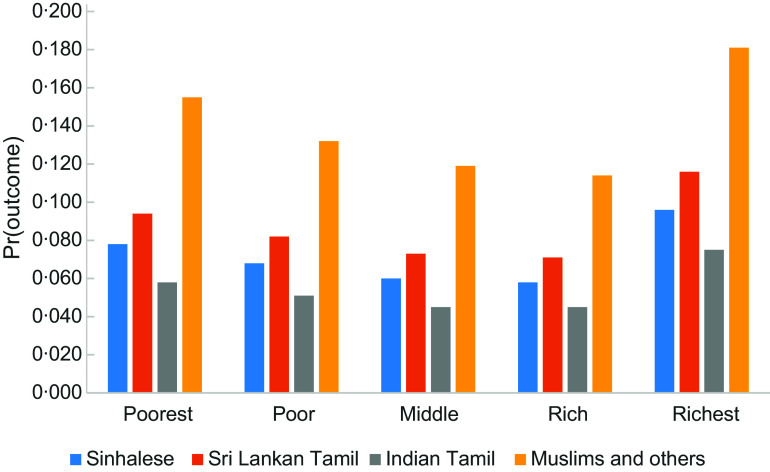
Figure 2 long description. Predicted probability of a child being stunted and having an overweight mother by wealth and major ethnic groups.

Figure 2 shows the predicted probabilities of a child being stunted and having an overweight mother by wealth and major ethnic groups. A U-shaped relationship was observed between household wealth and the likelihood of SCOWT pairs, with the highest probabilities in the richest and poorest households and the lowest in middle-income groups. Muslim women had the highest risk of DBM, while Indian Tamil women had the lowest, likely due to low rates of maternal overweight despite high child stunting.

## Discussion

Sri Lanka has demonstrated better health achievements compared to its South Asian peers and most LMIC. However, the country continues to face persistent childhood undernutrition alongside rising maternal overweight, obesity and NCD. The present study found evidence that the coexistence of child stunting and overweight mothers in Sri Lanka is 8·3 %, including 2·8 % of SCOB pairs. Comparable prevalence estimates have been reported in Bangladesh (4·10 %), Nepal (1·54 %), Pakistan (3·93 %) and Myanmar (5·54 %)^(31)^, as well as in Guatemala and Ghana^(7,32)^. These findings confirm that Sri Lanka exhibits a similar DBM pattern to those documented in other low- and middle-income settings. The present study highlights Sri Lanka’s emerging paradox of undernutrition and overnutrition within the same household.

When the child-related immediate factors are considered, dual burden pairs are, not surprisingly, associated with the predictors of stunting such as child age and LBW status consistent with previous studies conducted in LMIC^(8,32,33)^. There was a strong positive association between LBW and stunted child and other mother pairs. LBW status is also significantly associated with maternal BMI levels, as it significantly increases the odds of stunting across all maternal BMI categories, with the effect being strongest in children of underweight mothers. These findings are consistent with the existing literature^(14,15)^, highlighting the need for maternal and child nutritional interventions, particularly focusing on underweight mothers and LBW children.

Maternal factors indicate that advanced maternal age increases the risk of SCOWT pairs, while older mothers are less likely to be in the stunted/underweight mother pairs compared to young mothers. Evidence from multiple settings shows that overweight and obesity are more common among ageing women, and that older maternal age is associated with increased risks of LBW and stunting in infancy, as demonstrated in studies from Brazil, Guatemala, India, the Philippines and South Africa^(15,34,35)^. However, findings should be interpreted with caution, as the small sample size in the stunted–overweight subgroup may have resulted in wider CI and reduced precision of the estimates.

Short maternal stature was significantly associated with SCOWT pairs and SCOB pairs. This finding is consistent with the previous findings reported by Ferreira *et al.* ^(36)^ and Oddo *et al.* ^(37)^. Stunting appears as an intergenerational phenomenon that could transfer from mother to child^(15,36,37)^.

Mothers with short stature are potentially exposed to chronic diseases, and they may give birth to undernourished babies, who may later become overweight or obese, reflecting Barker’s DOHaD hypothesis^(12,15,36)^. The present study underscores the significant role of maternal stature in child undernutrition. Maternal education and employment showed limited association with DBM, though mothers with A-level education were 40 % less likely to have stunted children compared to those with only primary education, highlighting the link between lower maternal education and child stunting^(19)^. The lack of a significant association may reflect limited variability in maternal education in Sri Lanka, reflecting Sri Lanka’s high female literacy and overall educational attainment, as well as relatively small subgroup sample sizes that resulted in wide CI and reduced statistical power to detect significant differences.

The current study also found that double-burden pairs are more likely to be affected if they had a caesarean or other delivery than those born via normal delivery. This finding aligns with several studies conducted in various settings. Obesity has been linked to higher rates of caesarean and other delivery, with obese mothers also facing an increased risk of perinatal complications such as pre-eclampsia, gestational diabetes and high blood pressure^(38,39)^. Additionally, the mode of delivery may influence gut microbiota dynamics in stunted children^(40)^, warranting further investigation in Sri Lanka’s context.

Ethnicity was found to be a strong risk factor for the DBM at the household level as Muslim mothers have an elevated risk of being in a SCOWT or SCOB pair, and they are at the highest risk of being classified in the DBM category. This could be attributable to the different energy intakes among various ethnic groups that underpin their cultural eating habits: Muslims in Sri Lanka tend to have a higher energy intake, higher dietary diversity and eat more fat-rich foods^(41,42)^.

In addition, among other pairs studied, Indian Tamil mothers are more likely to be in stunted child–underweight mother pairs. The possible reason could be the highest malnutrition and nutritional deficiencies in the estate sector, where people often live with poor dietary diversity and socio-economic disadvantages^(41,43)^. This highlights the urgent need for public health initiatives, such as nutrition counselling and promoting healthy eating behaviours, at both the school and community levels. For example, a pregnant or lactating mother who is being overweight or obese could receive nutritional advice from the antenatal care and postnatal care platforms.

In 2020, the Sri Lankan government implemented a ‘Traffic Light Colour Coding System’ mandating that sugary products display colour codes based on their sugar, salt and fat content^(44)^. However, the system has certain limitations. Its scope is restricted to specific food categories, and there are information gaps and limited public understanding of the labelling system, particularly among different socio-economic groups^(45)^. Expanding the system’s scope and addressing these challenges could further help in reducing the risk of overweight and NCD.

In terms of other household factors, household wealth status did not have a consistent impact on the risk of double-burden pairs. This finding differs from some studies in Asian, African and Latin American countries, where it was found that DBM at the household level is predominantly associated with lower wealth index groups, reflecting economic disadvantage^(28,46)^. This suggests that the issue in impoverished households, particularly in Asian and African regions, is insufficient food quantity rather than poor food quality.

The relatively small sample sizes within some wealth subgroups in our dataset may have reduced the precision of the estimates, as reflected in the wide CI, potentially limiting our ability to detect significant gradients by socio-economic status. Further research using larger, nationally representative samples is warranted to better clarify wealth–DBM relationships in the Sri Lankan context.

When the residential factors are considered, paradoxical pairs are more common in urban settings. DBM pairs with overweight and obese mothers were more common in the urban sector (8·9 per cent and 4·3 per cent of households, respectively). However, this study also found that the regional effect was not strong, once all the covariates were adjusted to the final model. The lack of significance of the regional effect could be explained by the fact that immediate factors such as maternal stature, ethnicity and wealth may have taken up the regional effect.

Our findings highlight the multifaceted causes of household-level DBM and the need for holistic interventions addressing both under- and overnutrition. Policies should promote appropriate infant and young child feeding with nutrient-rich complementary foods, prevent excessive weight gain after the age of 2 years and improve access to affordable, healthy foods^(47)^.

### Strengths and limitations

This study uses the first national-level cross-sectional data collected after Sri Lanka’s war and civil conflict. To our knowledge, it is the first national study examining both the DBM among children and mothers and their coexistence within the same household.

However, several limitations should be considered when interpreting the findings. In particular, the cross-sectional design limits causal inference regarding the relationship between maternal and child nutritional status. Incomplete data on key anthropometric indicators, including height-for-age *Z*-scores and maternal BMI, may have introduced selection bias. Although standardised protocols were used, anthropometric measurements are subject to potential observer variation.

The absence of additional anthropometric measures, such as head circumference, mid-upper arm circumference and waist:hip ratio, may have reduced the precision of nutritional assessment. Moreover, standard BMI cut-offs may not accurately reflect adiposity among Sri Lankan adults, potentially underestimating the prevalence of overweight and obesity^(43)^. Other important behavioural and socio-environmental determinants of the DBM, including dietary intake, household food security, physical activity and caregiving practices, were not available in the dataset.

Finally, the exclusion of less prevalent mother–child nutritional pairings due to limited sample sizes may have led to an underestimation of the true household prevalence of the DBM. These limitations should be considered when interpreting the study findings.

## Conclusion

Our analyses have demonstrated Sri Lanka’s emerging DBM paradox with 8·3 % of households having a SCOWT pair and 2·8 % having a SCOB pair. Child age, LBW, maternal age, household size, mode of delivery, wealth status and province of residence were significantly associated with DBM, while Muslim ethnicity was associated with a higher likelihood of DBM at the household level.

With child undernutrition rates already stagnant, this dual challenge demands attention in national nutritional policies and interventions.

Further research is needed to explore the biological and socio-economic pathways linking diet to DBM, with disaggregated analysis by region, residential sector and ethnicity, particularly among Muslims, who experience high rates of overweight and obesity. Policy interventions should promote optimal infant and young child feeding, ensure adequate micronutrient intake to prevent early excessive weight gain and improve access to affordable nutritious food^(47)^. Finally, these findings underscore the need for early detection of both undernutrition and overnutrition to avert long-term nutritional challenges and their associated disease burdens.

## Supporting information

10.1017/S1368980026102742.sm001 Abeywickrama et al. supplementary material Abeywickrama et al. supplementary material

## Supplementary material

For supplementary material accompanying this paper visit https://doi.org/10.1017/S1368980026102742

## Table 1. Long description

The table presents a detailed analysis of the association between various factors and maternal BMI, categorized into thin, normal, overweight, and obese groups. It includes data on child stunting, child age, birth interval, sex, birth weight status, current breastfeeding status, child morbidity, maternal age, marital status, maternal education, maternal employment status, delivery mode, household size, wealth index, residential sector, ethnicity, province, and district. The table has 30 rows and 6 columns, with each row representing a different variable and its percentage distribution across the BMI categories. Notable trends include variations in child stunting, maternal education, and wealth index across different BMI categories.

Navigate back to Table 1..

## Table 2. Long description

The table presents data on the percentage of child-mother pairs categorized by various demographic and socio-economic characteristics. It includes columns for total cases, stunted child-underweight mother, stunted child-normal-weight mother, stunted child-overweight mother, stunted child-obese mother, and non-stunted child-normal-weight mother. The table also provides P-values for chi-square tests of association. Key characteristics include child age in months, sex, birth weight, child mortality, breastfeeding practices, maternal age and height, maternal and child education levels, household wealth index, household size, maternal employment status, and region of residence. Notable trends include variations in stunting and maternal weight across different socio-economic and demographic factors.

Navigate back to Table 2..

## Table 3. Long description

The table presents the results of a fully adjusted multinomial logistic regression model for child-mother pairs, adjusted for child, maternal, household, and residential characteristics. It includes covariates and categories such as child factors, maternal factors, and household factors. The table has 47 rows and 17 columns, with column headers including Intercept, Child Factors, Maternal Factors, and Household Factors. Each row provides adjusted relative risk ratios (aRRR) and 95 percent confidence intervals (CI) for different categories and subcategories. Notable trends include significant risk ratios for low birth weight, maternal height, and wealth index across different categories. The table highlights the independent associations of predictors with child-mother nutritional status pairs, with sampling weights applied across all analyses.

Navigate back to Table 3..

## Figure 1. Long description

The pie chart illustrates the distribution of child stunting status and maternal BMI among a sample of 3,470 individuals. The chart is divided into five segments. The largest segment, representing 68 percent, shows non-stunted children with normal-weight mothers, totaling 2,354 individuals. The second-largest segment, at 16 percent, depicts stunted children with normal-weight mothers, accounting for 559 individuals. The third segment, at 8 percent, represents stunted children with overweight mothers, totaling 288 individuals. The fourth segment, at 5 percent, shows stunted children with underweight mothers, amounting to 172 individuals. The smallest segment, at 3 percent, illustrates stunted children with obese mothers, totaling 97 individuals. The chart uses different colors to distinguish between these categories.

Navigate back to Figure 1..

## Figure 2. Long description

The bar graph compares the predicted probability of a child being stunted and having an overweight mother across different wealth categories and major ethnic groups. The x-axis represents wealth categories: Poorest, Poor, Middle, Rich, and Richest. The y-axis represents the probability of the outcome, ranging from 0.000 to 0.200. The graph includes four data series represented by different colors: Sinhalese in blue, Sri Lankan Tamil in orange, Indian Tamil in gray, and Muslims and others in yellow. Each wealth category contains four vertical bars, one for each ethnic group. Notable trends include higher probabilities for the Poorest and Richest categories, particularly for the Muslims and others group. All values are approximated.

Navigate back to Figure 2..
