# Supplementary material for: The double burden of malnutrition: coexistence of stunted children and overweight and obese mothers in Sri Lanka
Source: Public Health Nutr. 2026 Jun 1;29(1):e121. doi: 10.1017/S1368980026102742 (PMC13434500; doi:10.1017/S1368980026102742)
Supplement: Supplementary file 1 [file S1368980026102742sup001.docx]

Supplementary Figure 1:
